# DMSA-Net: a deformable multiscale adaptive classroom behavior recognition network

**DOI:** 10.7717/peerj-cs.2876

**Published:** 2025-04-30

**Authors:** Chunyu Dong, Jing Liu, Shenglong Xie

**Affiliations:** 1 School of Computing, Xijing University, Xi’an, Shaanxi, China; 2 School of Computer Science Technology, Xidian University, Xi’an, Shaanxi, China

**Keywords:** Classroom behavior recognition, Object detection, Attention mechanism, Feature fusion, IoU

## Abstract

In the intelligent transformation of education, accurate recognition of students’ classroom behavior has become one of the key technologies for enhancing the quality of instruction and the efficacy of learning. However, in the recognition of target behavior in real classroom scenarios, due to the use of wide-angle or panoramic images for image acquisition, students in the back row are far away from monitoring devices, and their subtle body movements such as the small opening and closing of the mouth (to determine whether they are speaking), fine finger operations (to distinguish between reading books or operating mobile phones) are difficult to recognize. Moreover, there are occlusions and scale differences in the front and back rankings, which can easily cause confusion and interference with target features in the detection process, greatly limiting the accurate recognition ability of existing visual algorithms for classroom behavior. This article proposes a deformable multiscale adaptive classroom behavior recognition network. To improve the network’s capacity to model minute behavioral phenomena, the backbone section introduces a deformable self-attention dattention module, dynamically modifying the receptive field’s geometry to enhance the model’s concentration on the region of interest. To improve the network’s capacity for feature extraction and integration of behavior occlusion and classroom behavior at different scales, a proposal has been put forward the Multiscale Attention Feature Pyramid Structure (MSAFPS), to achieve multi-level feature aggregation after multiscale feature fusion, reducing the impact of mutual occlusion and scale differences in classroom behavior between front and back rows. In the detect section, we adopt the Wise Intersection Over Union (Wise-IoU) loss as our loss criterion, augmenting the evaluation framework with richer contextual cues to broaden its scope and elevate the network’s detection prowess. Extensive experimentation reveals that our proposed method outperforms rival algorithms on two widely adopted benchmark datasets: SCB-Dataset3-S (the Student Classroom Behavior Dataset–https://github.com/Whiffe/SCB-dataset) and we created object detection dataset DataMountainSCB (https://github.com/Chunyu-Dong/DataFountainSCB1) containing six types of behaviors.

## Introduction

The incorporation of classroom teaching videos in recent times has unveiled vast opportunities for impacting student conduct (Gao et al., 2023). Classroom behavior patterns of students serve as crucial indicators of their learning progress, making the analysis and assessment of these behaviors instrumental in advancing teaching practices (Yusuf, Noor & Bello, 2024). However, with the accelerated advancement of educational informatization, how to accurately and quickly identify students’ key behaviors in the ever-changing classroom environment constitutes a formidable technical challenge in the domain of education, which urgently calls for a solution. The analysis of classroom behavior endeavors to uncover the underlying relationship between teacher instruction and student academic growth, facilitating self-reflection for both educators and learners and enhancing the overall quality of classroom instruction. Conventional approaches to analyzing classroom teaching behaviors rely heavily on subjective self-evaluations, direct oversight, and manual coding procedures (Akçapınar & Hasnine, 2022). These methodologies, however, are plagued by drawbacks including high subjectivity in coding practices, limited sample sizes, and significant time and labor investments, ultimately hindering their interpretability and scalability. Intelligent technology is harnessed for data acquisition and analytical endeavors, classroom behavior can be identified more timely and comprehensively, and the teaching and learning status of teachers and students can be understood, providing strong support for improving teaching quality. The adoption of deep learning, renowned for its robust feature extraction and autonomous learning process, has markedly enhanced the precision and speed of recognizing behaviors within classroom settings (Andrade, Delandshere & Danish, 2016).

The prevailing object detection frameworks are categorized into two fundamental groups: single-stage and two-stage detectors. The two-stage approach initially extracts regions of interest regions of interest (ROIs), followed by leveraging detection heads to categorize ROI features into target classes and refine their spatial locations. Representative two-stage detectors include Faster region-based convolutional neural network (RCNN) (Ren et al., 2017), Mask RCNN (Sudhanan, Rahuman & Roomi, 2025), and Cascade RCNN (Cai & Vasconcelos, 2018), among others. Conversely, single-stage detectors forgo the foreground screening step, directly classifying and regressing the feature maps extracted by the backbone network. Examples of popular single-stage detectors encompass single shot multibox detector (SSD) (Liu et al., 2016), RetinaNet (Lin et al., 2017), and the You Only Look Once (YOLO) series (Redmon et al., 2016; Zhang et al., 2023; Redmon & Farhadi, 2018; Bochkovskiy, Wang & Liao, 2020; Wang, Bochkovskiy & Liao, 2023; Wang, Yeh & Mark Liao, 2024; Van Etten, 2018). Generally, two-stage detectors exhibit superior accuracy at the expense of speed, whereas single-stage detectors, though typically inferior in accuracy, offer faster processing rates (Liu et al., 2024). Notably, numerous object detection methodologies are rooted in these two detector frameworks. Lately, visual techniques such as TransFormer (Tarasiou, Chavez & Zafeiriou, 2023), DETR (Zhao et al., 2024), and Mamba (Gu & Dao, 2023) have set new benchmarks in diverse domains, further advancing the state-of-the-art in object detection.

In actual classroom teaching environments, wide-angle or panoramic imaging is often used for image acquisition. The students seated in the rear rows are positioned at a considerable distance from the surveillance equipment, and their subtle body movements, such as the small opening and closing of their mouths (to determine whether they are speaking) and the fine operation of their fingers (to distinguish between flipping through books or operating mobile phones), are difficult to effectively extract features with sufficient discrimination. This leads to confusion of target features in the detection process, the remote positioning of students in the back row negatively impacts the efficacy of key processes such as feature extraction, fusion, regression, and classification, ultimately diminishing the precision and credibility of recognition outcomes. Consequently, there is an elevated risk of encountering missed detections and false positives. In addition, there are significant differences in the expression form, detail richness, and semantic level of the occlusion of front and back row action behaviors, as well as the characteristics of classroom behaviors at different scales. Using feature stacking or concatenation fusion methods will ignore the uniqueness and mutual promotion between features at different scales, resulting in insufficient information utilization.

Therefore, this article has designed a multiscale adaptive classroom behavior recognition network for rear micro-actions and occlusion sensitivity, this network architecture comprises three integral components: the backbone, which serves as the foundational layer; the neck, facilitating feature integration and enhancement; and the head, responsible for finalizing predictions through classification or regression. Through different strategies, we successfully overcame the challenges posed by small behavior targets about crucial tasks, including feature extraction, fusion, regression, and classification, thereby enhancing the overall efficiency and accuracy of the system, occlusion, and scale differences between behavior targets in actual classroom behavior recognition images during the detection process. In the backbone section, initially, we establish a comprehensive multiscale feature pyramid to efficiently extract multi-level features, enabling us to capture the intricacies associated with small behavior targets, and a deformable attention mechanism DAttention (Li et al., 2021) is introduced in the sixth layer of the pyramid, which can dynamically adjust the sampling position and attention weight. To enhance the model’s ability to attend to small behavioral targets, we incorporate a methodology that facilitates the extraction of more precise and accurate feature representations. To alleviate the influence of large-scale differences in the target ranking, neck further extracts and integrates features from multiple scales, proposing the Multiscale Attention Feature Pyramid Structure (MSAFPS), which fuses features from different scales to augment the model’s capacity and effectiveness to capture information at different scales in the image, effectively fuse features at different levels, and reduce the impact of mutual occlusion and scale differences in classroom behavior between front and back rankings. The head section of the network serves as the culmination point, where the extracted features are transformed and mapped onto the final output space, ultimately yielding the network’s prediction result. We employ a refined approach to quantify the degree of congruence between the predicted bounding box and the actual bounding box, thereby enhancing the accuracy of the network’s predictions, this article uses Wise-IoU to introduce richer contextual information to enhance the comprehensiveness of the evaluation. Introducing weights and considering surrounding area information, reduces the low quality of captured images and improves the robustness of the network. Our proposed DMSA net has the following advantages:

(1) The integration of the DAttention module, leveraging both deformable convolution and self-attention mechanisms, dynamically adjusts sampling positions and attention weights, enabling the model to prioritize smaller behavioral targets within classroom scenes. This enhanced focus facilitates the extraction of more precise feature representations, ultimately boosting the model’s ability to accurately model targets.
(2) Propose a novel MSAFPS, combined with expected maximization deformable attention and weighted feature pyramid network, to improve feature quality and reliability while achieving effective transfer and fusion between features at different levels. Realize noise reduction, purification, and effective fusion of features to obtain more robust feature expressions, reducing the impact of mutual occlusion and scale differences in front and back row classroom behavior.
(3) Introducing the Wise Intersection over Union (Wise-IoU) loss function, augmented with a dynamic non-monotonic focusing mechanism for bounding box localization, which enhances the model’s ability to precisely localize targets in complex scenes, Wise-IoU solves the problem of traditional IoU ignoring surrounding information and being sensitive to data quality. Introducing weights and considering surrounding area information, reduces the impact of low-quality target samples on the model’s generalization ability and improves the model’s detection ability for low-quality target samples.

The subsequent sections of this article are structured as follows: “Related Work” offers a comprehensive review of pertinent literature in classroom behavior recognition. In “Methods”, we delve into the specifics of our model and its constituent modules. Subsequently, “Results” presents ablation studies and comparative experiments to validate our model design, complemented by a visual analysis of detection performance. Lastly, “Conclusions” concludes our findings and outlines avenues for future research.

## Related work

### Classroom behavior recognition in deep learning

In recent times, the research landscape within the domain of computer vision has undergone profound transformations, transitioning progressively away from traditional approaches that hinged on manually crafted feature extraction and classifier training, towards an “end-to-end” learning paradigm that seamlessly integrates advanced machine learning and deep learning methodologies. Krizhevsky, Sutskever & Hinton (2017) introduced the groundbreaking deep learning model, AlexNet, achieving a notable milestone in the ImageNet Large Scale Visual Recognition Challenge. Subsequently, Girshick et al. (2014) devised the R-CNN detection framework, pioneering a two-stage approach: first, candidate regions are generated, followed by regression and training on these regions to yield bounding boxes accompanied by confidence scores. This approach was further refined through the introduction of Fast R-CNN (Girshick, 2015) and Faster R-CNN (Ren et al., 2017), both aimed at enhancing training speed and accuracy. In parallel, Liu et al. (2016) introduced the single shot multibox detector (SSD) algorithm, which, akin to the YOLO series, achieves the concurrent prediction of object categories and bounding boxes in a single pass.

Behavior recognition, as a pivotal area of investigation within the realm of computer vision, aims to automatically extract information about human actions or activities from video or image data, achieving efficient and accurate behavior classification and recognition. Gowda (2017) devised a method for classifying human behavior, incorporating deep belief networks. Their approach encompasses two networks: one employs an enhanced weber descriptor to capture features from target motion, while the other extracts image-based features, encoding spatiotemporal action information from frames and feeding these into a CNN for classification, thereby demonstrating exceptional classification results. In parallel, Carreira & Zisserman (2017) introduced a dual-stream inflatable 3D CNN, leveraging 2D network weights as pre-training for the 3D network. Moreover, the landscape of video action recognition continues to evolve, with advancements in methods leveraging long short term memory (LSTM) and generative adversarial neural networks (GAN) frameworks (Gu et al., 2021).

Given its robust capability in extracting intricate features (Krizhevsky, Sutskever & Hinton, 2017), deep learning technology has emerged as a formidable tool, that provides strong technical support for classroom behavior recognition. At present, research on classroom behavior mainly focuses on student facial expression recognition, class head-up rate, and recognition of abnormal classroom behavior. Wenchao et al. (2022) integrated the feature pyramid module into the SSD model, enhancing classroom behavior recognition efficiency. Tang et al. (2022) reframed action detection as a fine-grained classification task for action images, leveraging a weighted bidirectional feature pyramid network (BiFPN) alongside a feature pyramid structure to boost accuracy. Guo (2020) introduced a novel approach to identifying students’ head-up rates in classrooms, developing a method to extract salient facial features. Through a multi-task CNN, they demonstrated the effectiveness of detecting students’ head-up rates. Jisi & Yin (2021) combined spatial affine transformation networks with CNNs to extract richer features, fusing spatiotemporal features *via* a weighted sum method and employing an enhanced softmax classifier for classification and recognition. Abdallah, Elleuch & Guermazi (2021) presented a deep transfer learning-based method, initially pretraining the model on a facial expression dataset before utilizing the transfer model for classifying student behavior.

### YOLO application in classroom behavior recognition

The YOLO detection algorithm constitutes a one-stage, deep learning-grounded approach, which continuously adjusts the position and category of bounding-box in the output layer through regression. After a picture is input into YOLO, the backbone feature extraction network will continuously perform convolution operations on the image, repeatedly stacking and extracting features through the bottom three feature layers, and then using the obtained features for prediction. Finally, the final prediction box is calculated and output based on the three prediction results.

YOLO has emerged as a preeminent target detector, renowned for its optimal balance of speed and accuracy (Wang, Yeh & Mark Liao, 2024), and has garnered widespread adoption in educational research. Liu, Ao & Hong (2021) advanced YOLO-v3 by integrating cascaded, refined RFB modules, bolstering feature extraction, and harnessing shallow information for heightened small target recognition. To mitigate character occlusion in crowded classrooms, they substituted the SE-Res2Net module for Darknet-53’s ResN module, enabling multi-layer feature reuse. Zhang et al. (2020) enhanced YOLO-v3 with an attention mechanism, facilitating targeted training of student behavior characteristics, and achieved accurate recognition using the SICAU classroom dataset. Ren & Yang (2021) presented YOLOv4 Bi, a refinement of YOLOv4, further elevating its feature extraction prowess. Meanwhile, Hu et al. (2022) leveraged power IoU loss in YOLOv5, refining classroom behavior detection accuracy.

Liu et al. (2024) proposed an improved algorithm based on YOLOv8n to address the challenges of occlusion and small object detection in classroom behavior recognition. By introducing a BRA module to enhance the capture of fine-grained, global, and contextual information, the algorithm solves the occlusion problem. At the same time, the addition of a TODL module optimizes the ability to detect small objects, especially the behavior recognition of students in the back row, which improves the recognition performance in complex classroom scenarios.

Chen, Zhou & Jiang (2023) introduced an optimized YOLOv8 model tailored for classroom detection. They devised a novel C2f_Res2block module, blending elements from Res2Net and YOLOv8, which was subsequently integrated into the YOLOv8 framework alongside MHSA and efficient multiscale attention (EMA). Evaluations revealed superior detection performance on classroom datasets, outperforming the baseline YOLOv8.

Although the YOLO network has achieved some results in classroom behavior recognition, there are still some issues that may reduce the accuracy and reliability of recognition due to the small classroom behavior goals of students in the back row (such as their speaking behavior, phone usage behavior, *etc*.), as well as the occlusion and scale differences between students in the front and back rows. This article proposes a multiscale adaptive classroom behavior recognition network for detecting small behaviors and occlusion in the back row, which has been validated on multiple datasets and achieved good results.

## Methods

### Overall architecture

The latest iteration of the YOLO series, YOLO v10 (Wang et al., 2024), originates from the innovative efforts of Tsinghua University, marking a significant advancement in the series. YOLO v10 pioneers as the inaugural real-time, end-to-end object detection model within the YOLO series, and its network structure inherits from YOLOv8 (Gallagher, 2024). It addresses for the first time the issue of end-to-end deployment of YOLO hindered by post-processing dependencies with non-maximum suppression and proposes a consistent dual allocation strategy for training YOLO without NMS. The specific approach is to introduce two parallel output heads, which are used for one-to-one and one-to-many label allocation, respectively. The one-to-many branch in the training stage provides rich supervision signals, which helps the model learn. In the inference stage, only one to one branch is used for prediction, avoiding the computational overhead of NMS. To ensure that the supervision signals of the two branches are as consistent as possible during the training process, YOLOv10 adopts the consistent matching metric (CMM) standard, which helps reduce the supervision gap between the two branches and improve the overall performance of the model. In addition, YOLOv10 proposes an overall efficiency accuracy-driven model design strategy, which comprehensively optimizes various components of YOLO, mainly including: (1) Simplifying the architecture of the classification head and reducing computational complexity. (2) Decoupling space reduction and channel addition operations to improve the efficiency of downsampling. (3) Sort the stages based on their intrinsic rank to reduce redundancy and improve efficiency. (4) Use large convolutional kernels and partial self-attention modules to enhance the global representation capability of the model while maintaining low cost.

**Figure 1:**
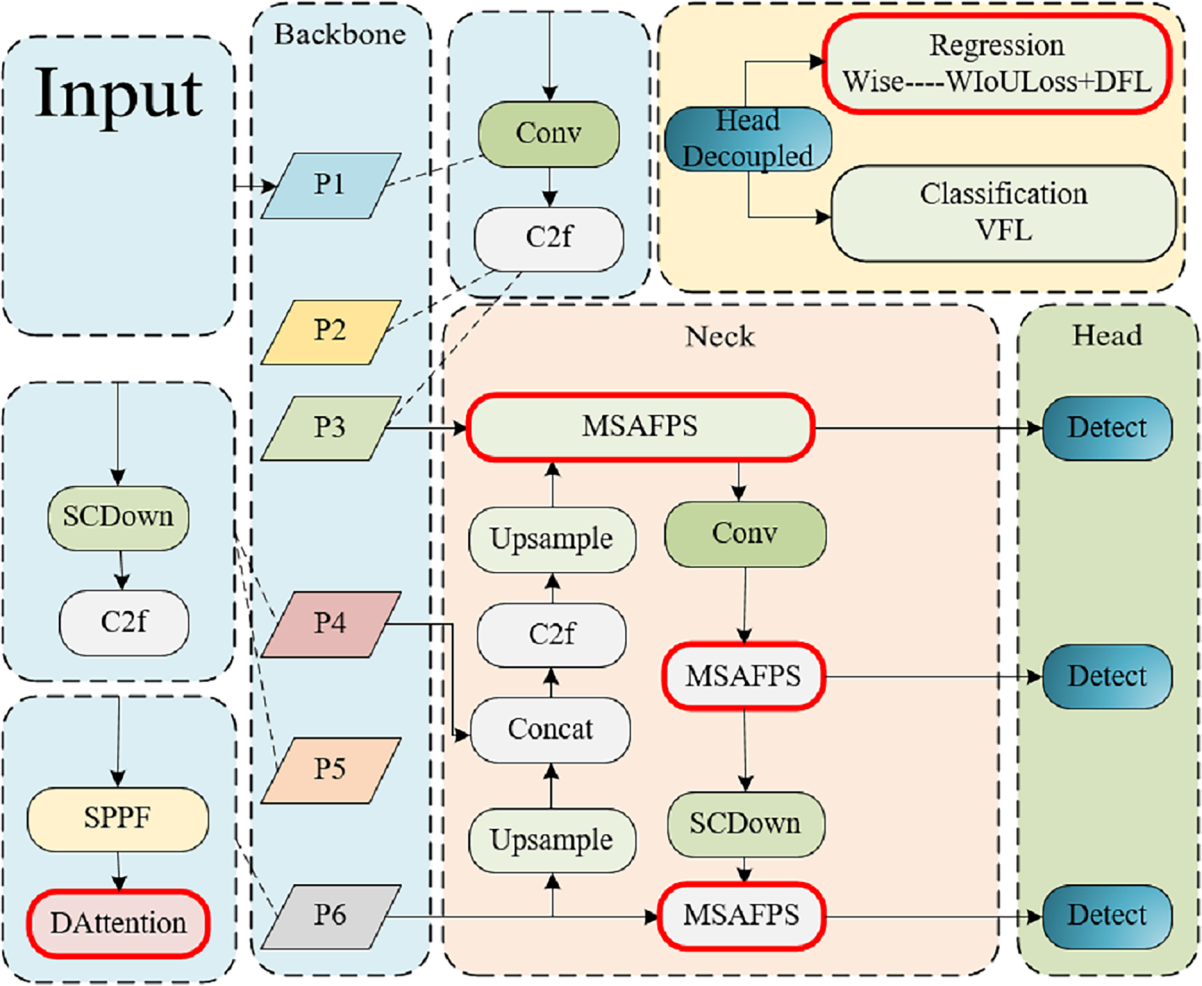
An illustrative overview of the DMSA net architecture highlights the integration of the DAttention mechanism within the backbone, facilitating the establishment of a six-tiered feature pyramid for comprehensive multiscale feature extraction. Neck designed the MSAFPS module to further achieve feature extraction and integration. The incorporation of the Wise-IoU loss function in the Head component enhances the precision with which target positions and shapes are characterized during localization and classification tasks.

We proposed the DMSA Net network based on YOLOv10, which also consists of three parts: backbone, neck, and head. As shown in Fig. 1, backbone constructs a six-layer feature pyramid using a CNN network (Pishchulin et al., 2016) to extract information with different scales and features. Layers 1–3 are composed of Conv modules and C2f modules, while layers 4 and 5 are composed of SCDond modules and C2f modules. The sixth layer incorporates the DAttention module, facilitating precise modeling of long-range dependencies and occlusion scenarios in classroom behavior analysis within intricate environments. Subsequently, at the neck layer, the feature maps undergo advanced extraction and fusion, leveraging the MSAFPS module for multilevel feature integration post-multiscal feature blending. The head layer then processes neck derived features to execute object detection, entailing the prediction of target locations and categories. In regression tasks, the Wise-IoU loss function enhances precision in characterizing target position and form, ultimately delivering refined network outputs comprising bounding box coordinates and category probabilities.

### Backbone

The backbone serves as the core component of the network, tasked with extracting essential features from input imagery for subsequent elaborate processing and in-depth analysis. It has multiple layers and parameters, which can extract advanced feature representations of images.

#### Backbone overall structure

**Figure 2:**
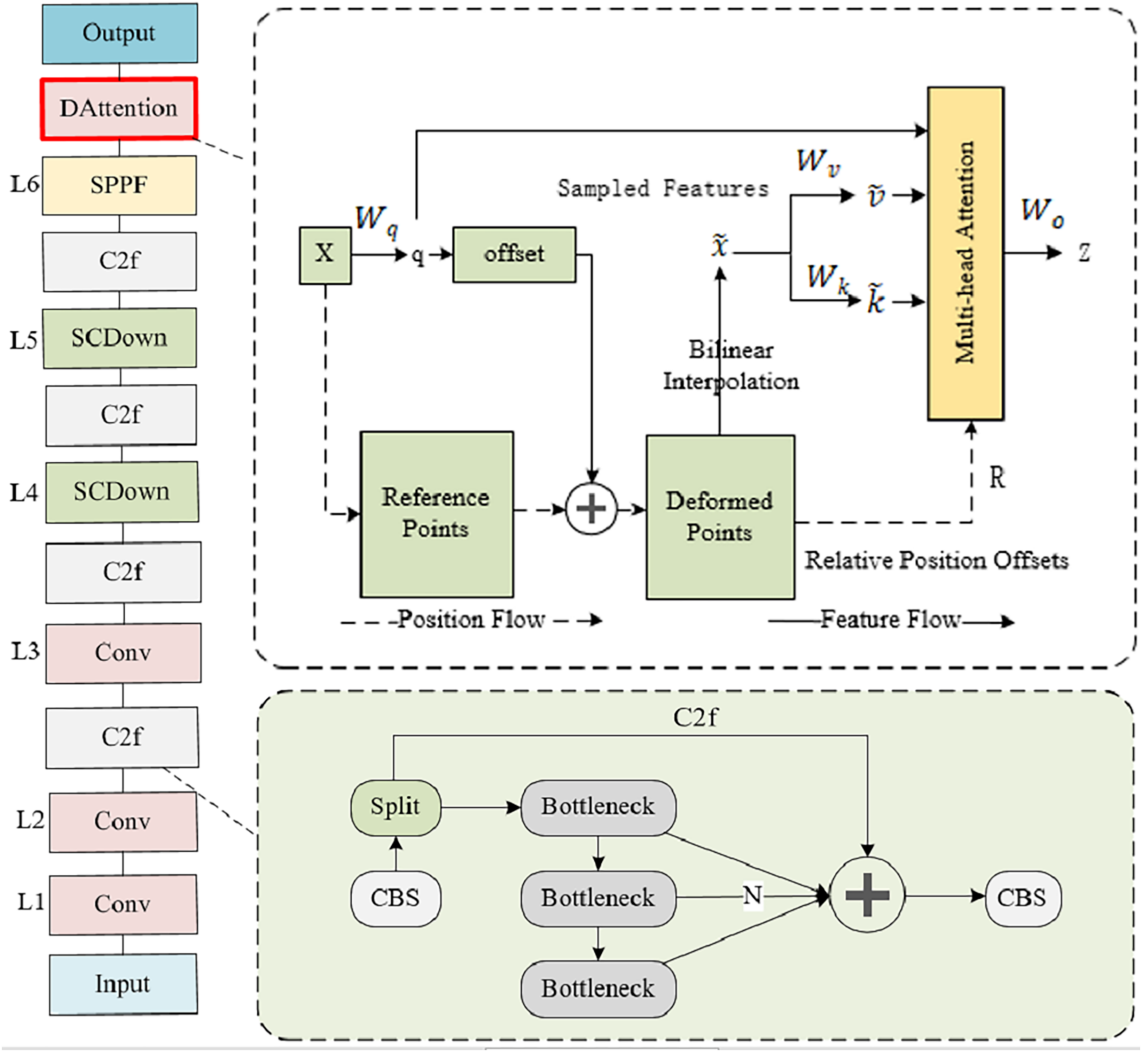
Backbone structure. Layer 1 consists of Conv modules, Layers 2–3 consist of Conv and C2f modules, and Layers 4–5 consist of SCDond and C2f modules. Layer 6 is composed of SPPF and DAttention modules.

Feature extraction plays a crucial role in classroom behavior recognition scenarios. However, in real classroom environments, image capture is mostly done with wide-angle and panoramic lenses, and the behavior in the back row of the classroom (such as speech judged by mouth shape, whether using a phone, *etc*.) has smaller targets, which poses great challenges for feature extraction. To overcome these challenges, DAttention was introduced in its backbone phase and a feature pyramid was constructed, as shown in Fig. 2. The initial layer, Layer 1, performs convolutional operations on the input image to extract foundational, low-level feature information, while Layer 2–3 consists of Conv and C2f modules. Layers 4–5 are composed of the SCDown module and the C2f module. Layer 6 consists of the SPPF module and the DAttention module.

#### Deformable attention mechanism

The core of backbone is the introduction of deformable attention machines. The DAttention mechanism dynamically adapts both the sampling position and attention weight, enabling a more flexible and contextually aware focus on salient features, enabling the model to more accurately focus on small target behaviors at long distances, enhancing the modeling ability of behavioral features in real classroom environments. By introducing this strategy, the backbone layer in this article can effectively extract robust features that can express objects of different scales from classroom behavior images, providing strong support for subsequent object detection tasks. DAttention represents a streamlined and effective deformable self-attention module, offering a lightweight yet powerful approach to modeling contextual relationships, which can also be seen as a spatial adaptation mechanism that allows models to dynamically adjust their attention focus while processing data. The traditional attention mechanism usually focuses intensively on the information of all grids during sampling, Meanwhile, the deformable self-attention mechanism enhances the model’s ability to precisely pinpoint and capture the salient features of objects within the data. While paying more attention to the classroom behavior target area, extracting more refined and accurate feature representations, and improving the modeling ability and feature expression ability for distant targets.

Input feature map $x \in {R^{H*W*C}}$, the purpose is to ascertain the displacement of each designated reference point, linearly project the feature map onto the query marker, expressed as $Bq = X{W_q}$, the reference points on the road are obtained by downsampling with proportional coefficients and generating grids. Given the input query, the offset network generates $\Delta p$, which is then represented as $\Delta p = {\emptyset _{offset}}(q)$. Subsequently, $\Delta p$ is added to the reference points to derive the offset position information. This information is then utilized to sample the deformed reference points through bilinear interpolation, yielding x, k, and v values:

(1) $$x = \phi (x;p + \Delta p)$$

(2) $$k = x{W_k}$$

(3) $$v = x{W_v}$$

Adding relative position encoding to calculate multi-head attention:

(4) $${z^{(m)}} = \delta \left( {{q^{(m)}}{k^{(m)t}}/\sqrt d + \phi (B;R)} \right){v^{(m)}}.$$

The deformable attention module exhibits an improved capability in capturing the intricate relationships between feature points across varying scales, enhancing its ability to represent multiscale features. By predicting an offset, the attention distribution is no longer fixed, but can adaptively change according to the characteristics of the data. This methodology allows the network model to prioritize and attend to critical feature information within classroom behavior imagery, particularly emphasizing the often overlooked small target behaviors in the rear. While minimizing redundant computations beyond simply broadening the receptive field. Deformable self-attention uses multiple sets of deformable sampling points to determine the important regions of the feature map and models the relationships between different features of classroom behavior based on these important regions. Reduce the influence of irrelevant background, non-classroom behavior, and occlusion between behaviors to improve the expressive ability of features.

### Neck

The neck, as an intermediary layer bridging the backbone and head, fulfills a pivotal role in resizing or modulating the dimensionality of backbone derived features to seamlessly align with task-specific needs. Additionally, it oversees the fusion of multiscale feature maps, subsequently relaying these enriched features to the prediction layer for further processing.

#### Multiscale attention feature pyramid structure MSAFPS

**Figure 3:**
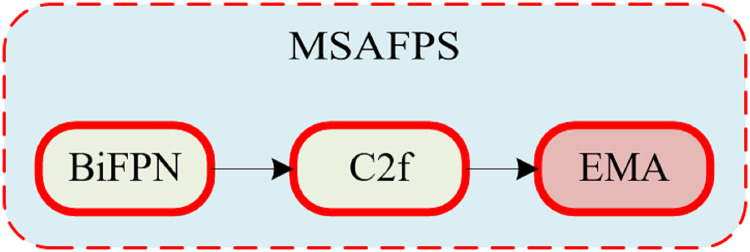
Multiscale attention feature pyramid structure MSAFPS structure.

In classroom behavior recognition tasks, given the spatial distribution of student objects within the classroom environment, there is a significant difference in classroom behavior between the front and back rows. Previous feature fusion methods have difficulty accurately distinguishing the significance of features spanning various scales, and some scales of features may be more important than others, resulting in a greater impact on the results and an uneven contribution of these features to the outcome. To tackle the aforementioned obstacle, this article proposes a multiscale attention feature pyramid structure called MSAFPS, as shown in Fig. 3, which enables efficient fusion of high and low-level features between different stages, thereby achieving simultaneous fusion of deep, shallow, and multiscale features. This mechanism can achieve denoising, purification, and effective fusion of features, thereby obtaining more robust feature expressions. The MSAFPS structure enables effective transfer and fusion between features at different levels. Specifically, BiFPN (Girshick et al., 2014) adjusts the importance of features on different paths through dynamic feature weighting, selectively fuses features, and achieves bidirectional feature propagation through multiple output connections to reduce information loss and propagation delay, thereby achieving efficient fusion between features at different levels. By utilizing EMA (Krizhevsky, Sutskever & Hinton, 2017) to maximize the expected value of attention weights, the model can emphasize pivotal information within the features while evening out the weight distribution, mitigating the impact of disruptive noisy features, and ultimately refining the output features.

BiFPN, a feature fusion network tailored for object detection, enhances both accuracy and efficiency *via* multilevel feature integration and dynamic weight assignment. It employs a bidirectional pyramid architecture, adeptly addressing information bottlenecks and feature distortions in conventional feature pyramids through adaptive fusion and selection mechanisms. Additionally, BiFPN incorporates cross-level connections and multiscale fusion, further bolstering the performance of object detection tasks.

**Figure 4:**
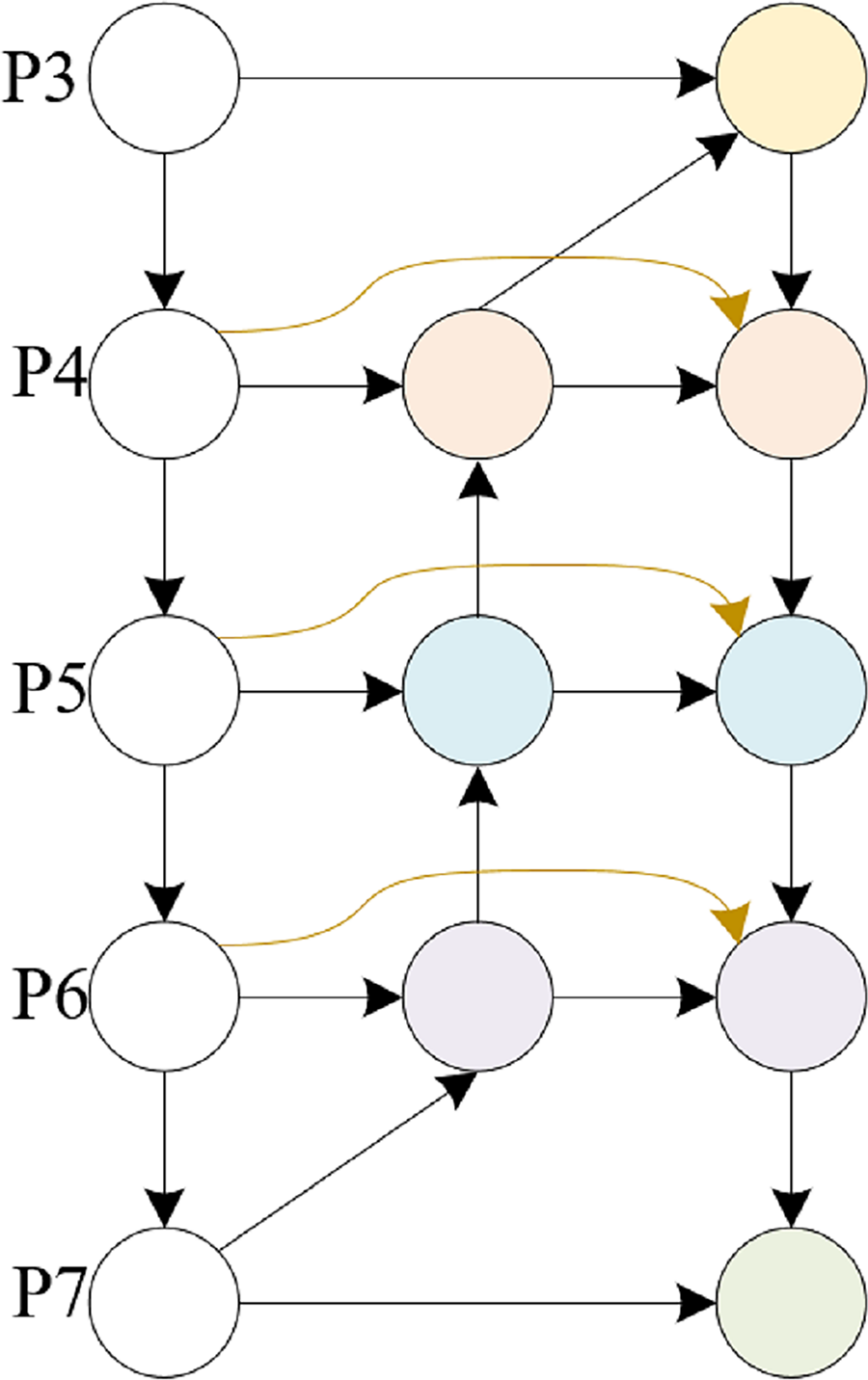
BiFPN structure.

**Figure 5:**
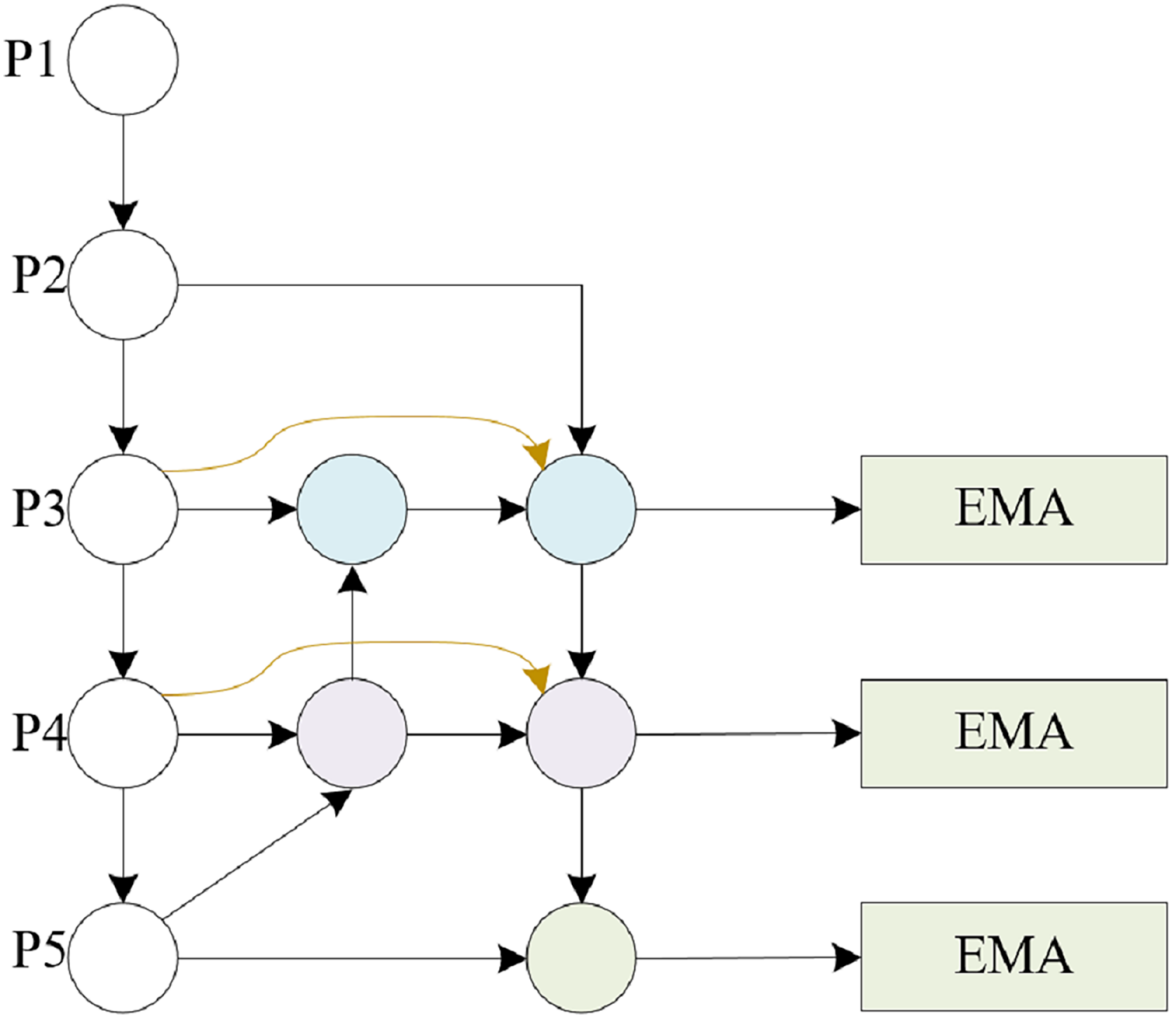
MSAFPS structure.

This article is based on the BiFPN structure and combines the feature input and detection head in this article to modify the BiFPN structure. The detection heads in this article only have three, so we further modified the five output detection heads of a normal BiFPN to support the detection of the three heads in this article. The detailed structure is shown in Figs. 4, 5.

**Figure 6:**
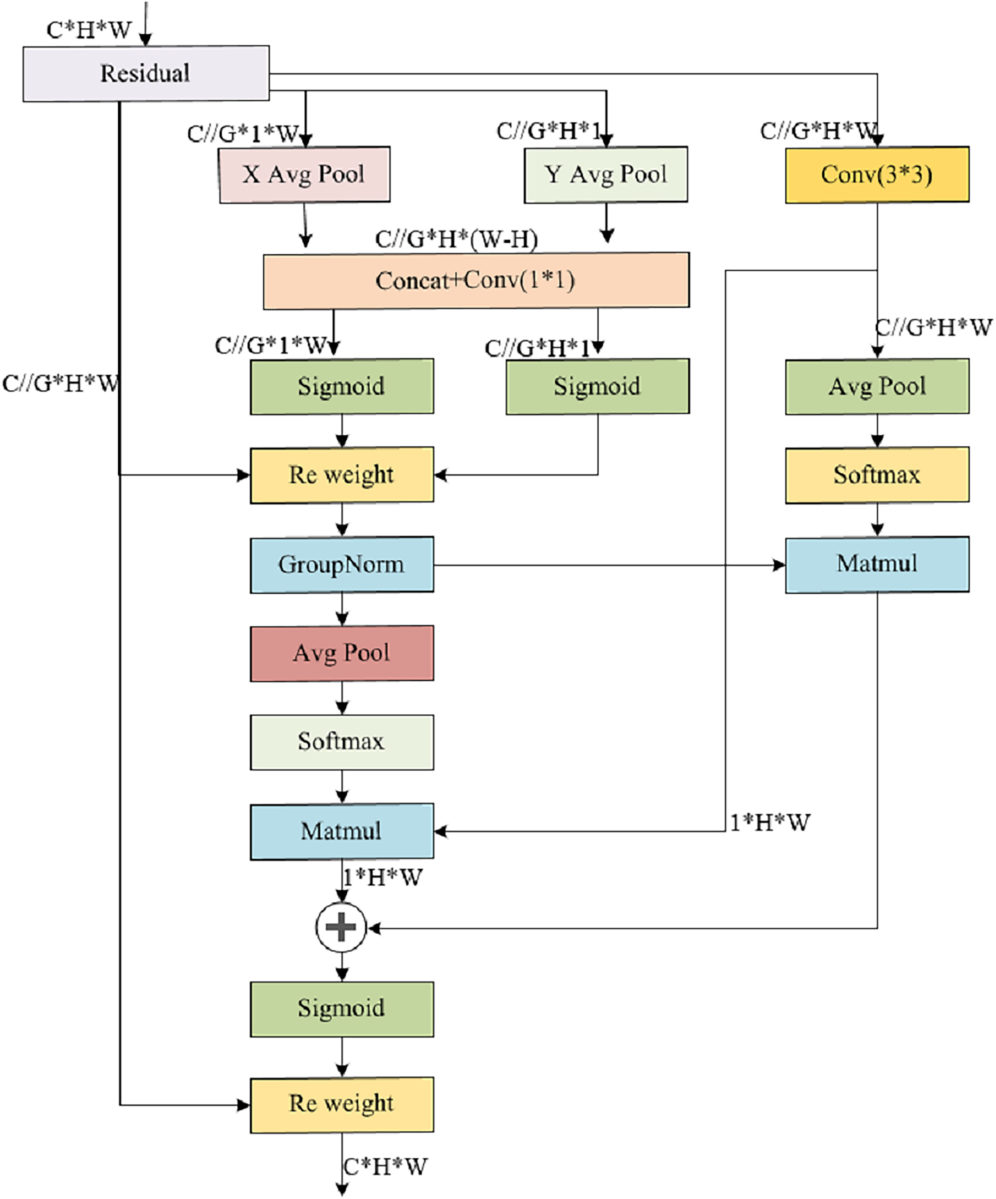
Illustrates the EMA structure, where C denotes the channel count within the feature map, H signifies its height, and W represents its width. The following grouping, X represents the resulting feature map, while X Avg Pool and Y Avg Pool indicate the application of one-dimensional horizontal and vertical global pooling respectively.

The EMA module strategically balances information preservation across channels while minimizing computational burden. It innovatively reorganizes channels into batch dimensions and partitions channel dimensions into sub-features, fostering a balanced distribution of spatial semantic features within each group. Notably, EMA not only encodes global information into parallel branches to refine channel weights but also fosters cross-dimensional interaction between the outputs of these branches, capturing intricate pixel-level relationships. Employing a parallel subnet architecture, EMA leverages three parallel paths to derive attention-weighted descriptors for grouped feature maps, emphasizing both multiscale features for holistic and detailed target understanding, and cross-spatial information aggregation across varying dimensions to grasp complex positional relationships among four target types. This intricate interplay results in richer aggregated features. The module integrates 1 × 1 and 3 × 3 branch outputs, harnesses two-dimensional global average pooling, and applies softmax-based linear transformations. By multiplying matrix dot products, the first spatial attention map emerges. Subsequently, the global spatial information from the 3 × 3 branch is encoded and synergistically fused with the 1 × 1 branch, yielding the second spatial attention map. The union of these two spatial attention weights captures the global context, and the final output dimensionally mirrors the input feature map. The detailed structure is shown in Fig. 6.

EMA provides channel and cross-spatial information exchange, which can adjust the focus on feature maps according to the scale requirements of each detection head. The BiFPN network efficiently integrates multi-level features, thereby enhancing the precision and swiftness of object detection. Our novel MSAFPS approach harmoniously fuses expected maximum attention with a weighted feature pyramid network, fostering improved feature quality and reliability, while facilitating seamless transfer and fusion across various feature levels.

### Wise-IoU loss

The definition of the loss function for bounding box regression (BBR) holds paramount importance in object detection, as its optimization can lead to substantial enhancements in model performance. Within the realm of object detection, scale differences and mutual occlusion between objects pose significant obstacles to improving the performance of regression models. Specifically, for small-scale targets, due to their small image area, the effective feature information extracted is relatively scarce, which greatly increases the difficulty of the regression model in accurately predicting their bounding boxes, resulting in a marked decline in prediction accuracy. On the contrary, for large-scale targets, although they contain rich feature information, due to their wide coverage of image areas, localization algorithms are more susceptible to adverse factors such as background noise, lighting changes, and interference from neighboring objects during processing, thereby weakening the accuracy of localization.

Scholars have introduced the CIoU loss function for boundary box regression, which incorporates three pivotal geometric aspects: overlap area between Ethe ground truth and predicted boxes, center point distance, and aspect ratio. This approach aims to align the predicted box more closely with the actual box, thereby refining regression accuracy. The calculation formula for the CIoU loss function is shown in Eqs. (5)–(7),

(5) $${L_{CIoU}} = 1 - IoU + {{{P^2}(b,{b^{gt}})} \over {{c^2}}} + av$$

(6) $$IoU = {{\left| {b \cap {b^{gt}}} \right|} \over {\left| {b \cup {b^{gt}}} \right|}}$$

(7) $$a = {v \over {(1 - IoU) + v}}$$

(8) $$v = {4 \over {{\pi ^2}}}\left[ {\arctan {{{w^{gt}}} \over {{h^{gt}}}}} \right] - \arctan {w \over h}.$$

The formula incorporates, b, the centroid of the predicted box; P, the Euclidean distance between their centers; C, the diagonal distance within the smallest enclosing rectangle of both boxes, serving as a weighting factor; V, quantifying aspect ratio consistency; IoU, the area intersection ratio between the actual and predicted boxes; the dimensions (width & height) of the actual box, W & H, respectively, the width and height of the predicted box.

While the CIoU loss function incorporates the aspect ratio of predicted and actual boxes, it neglects the challenge posed by low-quality samples within the training dataset. In real classroom environments, the diversity of student positions, changes in scale, and occlusion are major challenges. Low-quality examples are inevitably included in the training data, which can lead to regression errors and cause imbalanced training samples. These low-quality samples dominate the gradient and cause severe oscillations in the loss function, this, in turn, diminishes the model’s capacity for generalization. To solve such problems, reduce the impact of low-quality samples on gradients, and improve the learning ability of difficult samples such as occluded targets and rear-row student targets, this article uses Wise-IoU V3 instead of CIoU. Inevitably, the training dataset comprises low-quality samples. The established parameters of anchor boxes (such as distance and aspect ratio) may inadvertently heighten the network’s focus on these suboptimal samples, compromising generalization performance. To mitigate this, Wise-IoU V3 softens the penalties associated with these metrics and minimizes pre-training interference when anchor and target boxes exhibit high overlap, thereby enhancing the network’s generalization capabilities. The mathematical formulation of Wise-IoU V3 is detailed in Eqs. (9) through (12):

(9) $${L_{IoU}} = 1 - IoU$$

(10) $${R_{WIoU}} = exp\left[ {{{{{(x - {x_{gt}})}^2} + {{(y - {y_{gt}})}^2}} \over {{{(w_g^2 + h_g^2)}^*}}}} \right]$$

(11) $${L_{WIoUv1}} = {R_{WIoU}}{L_{IoU}}$$

(12) $$\beta = {{{L_{IoU}}} \over {\overline {{L_{IoU}}} }} \in \left[ {0, + \infty } \right)$$

(13) $$\gamma = {\beta \over {\delta {a^{\beta - \delta }}}}$$

(14) $${L_{WIoUv3}} = \gamma {L_{WIoUv1}}.$$

Herein, x and y denote the horizontal and vertical coordinates of the prediction box’s center, respectively, while w and h represent the width and height of the prediction box, respectively, ${x_{gt}}$, ${y_{gt}}$, ${w_{gt}}$, and ${h_{gt}}$ representing the spatial coordinates at the intersection of the median lines of the genuine box’s boundaries, as well as the width and height of the real box, ${w_g}$ and ${h_g}$. The width and height of the minimum closed box formed by the predicted box and the real box area. $\beta$ is the outlier, the larger its value, the worse the quality of the sample. The focus coefficient r is calculated from $\beta$, and the values of $\alpha$ and $\delta$ are 1.8 and 3. As the loss value escalates, the parameter r undergoes non-monotonic fluctuations. By adaptively modulating the gradient’s sensitivity to these subpar samples *via* r, we can expedite the network’s convergence process and bolster the model’s precision in localizing targets.

LIoU is dynamic, and as the model is trained, the threshold is automatically adjusted, and the criteria for discerning anchor box quality are rendered dynamic, underpinning a prudent strategy for gradient gain allocation. By tempering the dominance of superior anchor boxes and mitigating the detrimental gradients emanating from inferior samples, Wise-IoU achieves a nuanced, non-monotonic focus on intermediate samples. This adaptive approach fortifies the model’s capacity for generalization and elevates its overall performance metrics.

## Results

### Data set

We assessed the efficacy of our novel DMSA net architecture on two benchmark datasets: SCB-Dataset3-S (https://github.com/Whiffe/SCB-dataset) (Yang, Wang & Wang, 2023) and DataFountainSCB (Yang & Wang, 2023), thereby validating its performance across diverse data landscapes. The SCB-Dataset3-S dataset is an open dataset focused on the design of classroom behavior recognition tasks. This dataset was released in December 2024 and includes 5,015 images and 25,810 tags, with a focus on three behaviors: raising hands, reading, and writing. At least one target exists in each image, and most images contain more than one target. The detailed information is shown in Table 1.

**Table 1:** Units for magnetic properties.

Target classes	Target numbers
Hand-raising	11,207
Reading	10,841
Writing	3,762

The other dataset named DataMountainSCB (https://github.com/Chunyu-Dong/DataFountainSCB1) was created by ourselves. Table 2 lists the detailed information of the dataset.This dataset mainly focuses on six common behaviors of students in the classroom, including raising hands, reading, writing, playing with mobile phones, lowering heads, and lying on tables. Based on this, an image dataset was established, and nearly 2000 data points were successfully collected. The aforementioned dataset was partitioned into training, validation, and testing subsets, adhering to a 7:2:1 ratio, ensuring a balanced representation for model development, refinement, and evaluation, respectively.

**Table 2:** Detailed introduction to the DataFountainSCB dataset.

Target classes	Target numbers
Hand-raising	8,942
Reading	7,406
Writing	3,320
UsingPhone	172
BowingHead	964
LeaningTable	1,100

### Evaluation indicators and experimental environment

#### Evaluation indicators

To authenticate the proficiency of our proposed network in recognizing classroom behaviors, we conducted a comprehensive evaluation of its performance metrics, encompassing precision (P), recall (R), and mean average precision (mAP). The underlying mathematical formulations for calculating these indicators—accuracy, recall, and mAP—are outlined subsequently:

(15) $$precision = {{TP} \over {TP + FP}}$$

(16) $$recall = {{TP} \over {TP + FN}}.$$

Specifically, TP signifies the count of accurately identified classroom behavior instances, FP denotes the instances misclassified as classroom behavior, and FN reflects the number of actual classroom behavior goals that were overlooked by the model.

(17) $$mAP = {{\mathop \sum \nolimits_{n = 1}^N A{P_N}} \over N}$$

where in, n signifies the cumulative count of distinct categories, k denotes the number of detections performed, an AP serves as a metric to quantify the average precision achieved across each category.

The metric mAP50 captures the average precision at an IoU threshold of 0.5, while mAP50-95 encompasses a range of IoU thresholds from 0.5 to 0.95, averaging their respective AP values. This comprehensive approach transcends the constraints of single-category evaluations, offering a holistic view of the model’s performance across multiple classes. By comprehensively assessing detection results, mAP not only measures the effectiveness of object detection algorithms but also evaluates their stability and robustness in tackling multi-class scenarios. Its intuitive nature facilitates easy understanding and direct comparison, making it a prevalent evaluation metric in the field of object detection.

#### Experimental environment

The training regimen for our model entailed a comprehensive setup, with 500 epochs, a batch size of 4, 8 parallel processes, and an input image resolution of 640 × 640. For optimization, we opted for the SGD optimizer, complemented by a weight decay factor of 5e-x to counteract overfitting. Momentum was set at 0.937 to accelerate convergence. Additionally, we implemented Early Stopping, a mechanism that automatically halts training upon validation loss stabilization, ensuring our model achieved fundamental convergence.

Our model’s experimentation was conducted within an Ubuntu 20.04 environment, leveraging PyTorch 2.0.1, Python 3.9.19, and CUDA 12.2.79. The hardware setup featured an Intel (R) Core (TM) i7-9700KF CPU clocked at 3.60 GHz, complemented by an NVIDIA GeForce GTX 1050 Ti GPU. Processing 640 × 640 pixel images with three color channels, our approach comprises 385 layers, entailing 2.71 million parameters, achieving 8.4 GFLOPs of computational efficiency. The training protocol involved 500 epochs, resulting in a model size of 5.51 MB, optimized for the given task.

### Ablation experiment

#### Algorithm overall experiment

To solidify the efficacy of individual components within our proposed approach, we embarked on ablation studies, leveraging the SCB-Dataset3-S and DataFountainSCB datasets as benchmarks. These experiments were anchored against YOLOv10 as a foundational comparison, with the outcomes about the SCB-Dataset3-S detailed in Table 3, offering insights into the contribution of each module.

**Table 3:** SCB-Dataset3-S dataset ablation experiment.

DAttention	MSAFPS	Wise-IoU	mAP50(%)
X	X	X	73.1
$\surd$	X	X	73.5
X	$\surd$	X	73.8
X	X	$\surd$	73.7
$\surd$	$\surd$	X	74.2
X	$\surd$	$\surd$	74.1
$\surd$	$\surd$	$\surd$	74.5

The baseline, featuring a robust architecture with numerous convolutional and pooling layers within its backbone, attained a mAP50 score of 73.1%. This performance can be attributed to its efficient utilization of C2f structuring in convolutional layers, which not only enriched feature representations through additional skip connections and split operations but also mitigated computational overhead. While maintaining lightweight, they can obtain richer gradient flow information. These operations can achieve good results in natural scenes, but for classroom behavior recognition scenes, the background is complex, there are many small targets, and some objects are severely occluded, which still have certain limitations.

Therefore, we introduced the DAttention module on the baseline, which improved mAP50 by 0.4%. The fusion of CNN’s localized sensitivity with ViT’s global comprehension within the DAttention module allows the model to pinpoint salient areas with greater precision, thereby adeptly capturing spatial nuances within images and fortifying its capability to decipher intricate student behavior patterns even amidst lengthy distances or obstructions. After adding the MSAFPS structure, mAP50 increased by 0.7%, indicating that the combination of MSAFPS with expected maximum attention and weighted feature pyramid network improves feature quality and reliability while achieving effective transfer and fusion between features at different levels and scales. The incorporation of the Wise-IoU loss function led to a notable 0.6% elevation in mAP50, signifying its proficiency in accurately quantifying the overlap between predicted and ground truth bounding boxes, thereby minimizing the detrimental effects posed by suboptimal samples.

Incorporating both the DAttention module and the MSAFPS module into our approach led to a notable 1.1% boost in mAP50 over the baseline, demonstrating a synergistic enhancement that surpassed the incremental gains achieved by integrating each module individually, indicating that the two modules have good independence and can play a good role in different stages of detection. After adding the DAttention, MSAFPS, and Wise-IoU modules, the best results were achieved, with a 1.4% increase compared to the baseline, indicating that these modules may have a synergistic effect. They can better adapt to object detection tasks in complex scenes by coordinating and optimizing various aspects of the network, jointly improving the performance of classroom behavior image object detection tasks.

Consistent enhancements were observed in the DataFountainSCB dataset as well, as depicted in Table 4. Networks featuring overlapping blocks surpassed their single-module counterparts in performance. By incorporating three distinct modules, the model demonstrated adeptness in capturing spatial cues, comprehending multidimensional features, and dynamically fusing them, thereby mitigating the disruptive effects of intricate backgrounds. This holistic approach, devoid of any inter-module conflicts, culminated in an optimal model performance of 94.8%, underscoring the synergistic benefits of the proposed techniques.

**Table 4:** Ablation experiment on the DataFountainSCB dataset.

DAttention	MSAFPS	Wise-IoU	mAP50 (%)
X	X	X	93.3
$\surd$	X	X	93.8
X	$\surd$	X	94.0
X	X	$\surd$	94.1
$\surd$	$\surd$	X	94.4
X	$\surd$	$\surd$	94.6
$\surd$	$\surd$	$\surd$	94.8

#### DAttention module ablation experiment

To assess the efficacy of the DAttention module in various contexts, we performed ablation studies targeting distinct locations within the backbone network’s architecture. Given that layers 1 through 4 of this six-layered structure primarily facilitate the extraction of high-resolution image features, they were deemed less conducive for integrating attention mechanisms. Consequently, our focus shifted to experimenting with the 5th and 6th layers. The ablation outcomes, as reflected in Table 5 for both the SCB-Dataset3-S and DataFountainSCB datasets, offer valuable insights into the module’s performance.

**Table 5:** DAttention module ablation experiment.

Model layer	SCB-Dataset3-S		DataFountainSCB	
	mAP50	mAP50-95	mAP50	mAP50-95
Ours (No DAttention)	0.731	0.543	0.933	0.735
Layer5	0.733	0.555	0.936	0.758
Layer6	0.735	0.558	0.938	0.769
Layer5 + Layer6	0.732	0.551	0.932	0.732

Upon examination of the table, it is evident that omitting the DAttention module in our initial setup results in a baseline performance. Introducing the DAttention module exclusively in the sixth layer led to the most notable enhancement in detection accuracy. Conversely, appending it to the fifth layer individually or simultaneously with the sixth layer produced either modest gains or a decrease in performance, respectively. This observation aligns with the fact that deeper layers in the network architecture transition from encoding low-level details to higher-level semantic representations. As such, positioning the DAttention module in deeper layers, like the sixth, optimizes its capability to process complex semantic information. The expanded receptive field of the sixth layer, capable of encompassing richer contextual cues, further underpins this strategy’s effectiveness. DAttention dynamically adapts the receptive field’s dimensions to enhance feature extraction within the target region, thus refining target localization and recognition accuracy. While the fifth layer’s receptive field, albeit smaller than the sixth’s, suffices for the present dataset, integrating DAttention therein yields performance gains, albeit less pronounced than in the sixth layer. Simultaneous deployment across both layers risks extracting redundant features, potentially hindering detection performance by introducing noise. Additionally, this dual-layer approach significantly amplifies network complexity, augmenting computational demands and complicating training optimization, potentially leading to diminished overall performance and detection accuracy. Consequently, this study prioritizes integrating DAttention exclusively at the sixth layer.

#### Wise-IoU module ablation experiment

Based on the loss function, we conducted different comparative experiments on CIoU (Krizhevsky, Sutskever & Hinton, 2017), Wise-IoU V1 (Wenchao et al., 2022), Wise-IoU V2, and Wise-IoU V3. For the DataFountainSCB dataset, the results are shown in Table 6:

**Table 6:** IoU module ablation experiment.

Model	Precision	Recall	mAP50	mAP50-95
CIoU	0.724	0.824	0.933	0.752
Wise-IoU V1	0.824	0.859	0.935	0.76
Wise-IoU V2	0.839	0.862	0.936	0.762
Wise-IoU V3	0.846	0.874	0.941	0.769

The aforementioned results highlight Wise-IoU V3’s superior performance on the DataFountainSCB dataset in this study. This advantage stems from Wise-IoU unique approach of multiplying the IoU of the target box by a region weight, which dynamically adjusts based on the target’s position and scale. Employing Wise-IoU as an evaluation metric enhances the precision of target bounding box measurements and consequently boosts the overall detection algorithm’s performance. Notably, Wise-IoU V3 excels in handling classroom behavior images with low-quality samples due to this adaptive weighting mechanism. Conversely, Wise-IoU V1 attempted to address low-quality training examples through an attention-based bounding box loss, but geometric metrics like distance and aspect ratio inadvertently imposed harsher penalties on such samples, ultimately hindering the model’s generalization capabilities. An optimal loss function ought to mitigate the influence of geometric metrics’ penalties when anchor boxes align well with target boxes, while judicious intervention during training can bolster the model’s generalization prowess. Building upon distance metrics, distance attention underpins Wise-IoU V1’s two-tiered attention framework. Advancing further, Wise-IoU V2 introduces a monotonic focusing mechanism tailored for cross-entropy, strategically diminishing the loss contribution from straightforward examples. This strategy directs the model’s attention towards challenging instances, thereby enhancing classification performance. Extending this concept, Wise-IoU V3 establishes a dynamic quality partition criterion for anchor boxes, enabling it to devise the most fitting gradient gain allocation strategy in real time, tailored to the prevailing circumstances.

**Figure 7:**
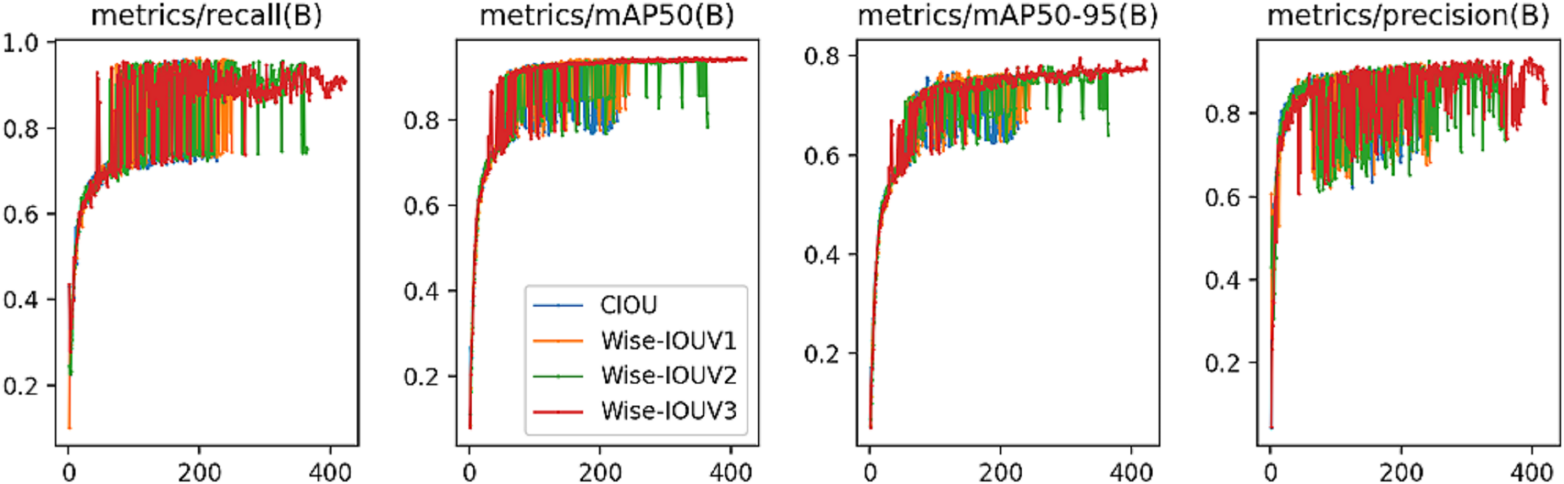
Experimental performance results of IoU module ablation comparison.

Figure 7 presents comparative curves for Precision, Recall, mAP50, and mAP50-95, revealing Wise-IoU V3’s consistently high performance throughout the training phase. Notably, after a certain iteration threshold, Wise-IoU V3’s performance gradually stabilized, ultimately achieving the peak values among the evaluated metrics.

### Comparative experiment

#### SCB-dataset3-S result

To substantiate the efficacy of our proposed approach, we conducted comparative experiments on the SCB-Dataset3-S dataset, juxtaposing it against other prevalent classical methodologies, the results are shown in Table 7. For the entire classroom behavior object detection, the mAP50 method proposed in this article improves by 3.4% compared to YOLOv5, 2.1% compared to YOLOv8, and 1.45% compared to YOLOv10. The model demonstrates robust performance even in scenarios where the overlap between predicted and actual bounding boxes is minimal, underscoring its resilience. Additionally, MAP50:95 registers a 1.2% enhancement over YOLOv10, affirming the model’s consistently high performance across varying low overlap union (LOU) thresholds.

**Table 7:** Comparative experiments based on SCB-Dataset3-S.

Models	P (%)	R (%)	mAP50 (%)	mAP50:95 (%)	Params (M)	GFLOPs	FPS
Faster-RCNN	51.3	54.8	65.4	39.7	15.84	13.56	19.93
RetinaNet	54.5	56.7	68.7	40.5	10.98	12.74	12.49
YOLOv5n (Thuan, 2021)	68.1	67.5	71.1	48.3	3.46	8.4	23.47
YOLOv8n	67.9	67.7	72.4	55.0	3.01	8.1	28.32
YOLOv10n	68.8	68.1	73.1	55.4	2.8	7.9	31.6
rtdetr-l (Zhao et al., 2024)	55.7	57.4	68.9	50.8	31.99	103.4	12.75
CSB-YOLO (Zhu & Yang, 2024)	67.35	65.48	70.3	51.6	1.86	6.2	36.41
Ours	69.6	68.8	74.5	56.6	2.61	7.4	35.12

Table 7 showcases the detection prowess of the method presented herein, demonstrating its proficiency in identifying diverse classroom behavior targets, regardless of type, size, or orientation. Notably, it excels in intricate scenes and exhibits remarkable resilience to varying lighting conditions, occlusions, and background distractions. Compared with the CSB-YOLO network, mAP50 increased by 4.2%, but Params and GFLOPs were larger.

The introduction of the DAttention module into our backbone network significantly contributes to improved detection accuracy. This module leverages deformation sampling points to adaptively reshape and resize the receptive field, thereby refining the feature extraction of target regions and bolstering the model’s modeling prowess for classroom behavior images. Additionally, the MSAFPS network facilitates seamless feature transfer and fusion across diverse levels and scales in the feature integration and extraction stages. Furthermore, the Wise-IoU loss function, employing a dynamic non-monotonic FM-based gradient gain allocation strategy, further enhances the overall performance. By introducing weights and considering surrounding information, it balances the punishment of detecting anchor boxes and enhances the model’s generalization performance. Through this innovative approach, our model has achieved significant performance improvements in regression and localization, especially when dealing with targets with scale differences, demonstrating stronger robustness and accuracy.

#### DataFountainSCB

Table 8 presents a comparative analysis of our proposed method against leading advanced techniques on the DataFountainSCB dataset. Notably, our approach demonstrates improvement on this benchmark as well.

**Table 8:** Comparative experiments of different models in DataFountainSCB.

Models	P (%)	R (%)	mAP50 (%)	mAP50-95 (%)	Params (M)	GFLOPs	FPS
Faster-RCNN	69.5	68.4	84.9	64.7	15.83	13.54	19.92
RetinaNet	72.4	71.5	87.2	66.2	10.96	12.76	12.48
YOLOv5n (Thuan, 2021)	84.6	84.2	91.5	72.1	3.48	8.5	23.50
YOLOv8n	87.8	85.0	92.4	73.7	3.03	8.2	28.31
YOLOv10n	91.1	87.8	93.3	73.6	2.84	7.8	31.59
rtdetr-l	73.5	72.4	87.4	67.5	31.97	103.1	12.77
CSB-YOLO	90.7	85.8	91.4	75.7	1.84	6.1	36.52
Ours	92.1	88.3	94.8	79.1	2.6	7.3	35.67

The aforementioned scenarios pose significant challenges to object detection due to factors like dense arrangements, varied orientations, occlusions, and small targets, amidst complex backgrounds, diverse environments, and extensive variations in scale and overlap. Our proposed method adeptly addresses these challenges by dynamically adjusting the receptive field’s size and shape, efficiently fusing and disseminating contextual and salient features across multiple scales, and resolving issues such as bounding box overlap *via* tailored loss functions. Consequently, it enhances the capability to accurately recognize behaviors within classroom settings.

The performance of distinct neural network algorithms varies notably when confronted with intricate scenes, images featuring minuscule target proportions, and densely populated sample distributions. Among them, the method proposed in this article has demonstrated good detection ability in multiple scenarios, especially when dealing with complex backgrounds, occluded objects, small targets of students in the back row, and some samples with low quality. However, no algorithm can achieve perfect detection performance in all scenarios, and each algorithm has its advantages and limitations. Hence, for practical deployment, selecting algorithms tailored to specific scenarios and requirements is crucial, often necessitating tailored optimizations and enhancements to meet performance targets.

## Conclusions

To accurately recognize the subtle movements and behaviors of students in the back row in real classrooms, such as the small opening and closing of the mouth (to determine whether they are speaking) and the fine manipulation of fingers (to distinguish between flipping through books or operating mobile phones), this article proposes an DMSA Net, which includes three parts: backbone, neck, and head. Constructing a multiscale feature pyramid within the backbone stage and incorporating a DAttention module serves to preserve both comprehensive background details and prominent feature information, enhancing overall detection capabilities. But also dynamically adjusts sampling positions and attention weights, this approach enables the model to prioritize smaller behavioral targets within the image, facilitating the extraction of finer, more precise feature representations, thereby improving detection accuracy. To enhance feature extraction and fusion across multiple scales, and tackle the challenge of behavioral occlusion, we implement a strategy that leverages the strengths of multiscale analysis and effective occlusion handling, neck proposed the MSAFPS, which combines expected maximum attention and weighted feature pyramid network to improve feature quality and reliability while achieving effective transfer and fusion between features of different levels and scales. By incorporating Wise-IoU loss in the head section, DMSA Net gains the ability to more precisely gauge the similarity between predicted and actual bounding boxes. This advancement not only boosts detection accuracy but also enhances the model’s resilience in complex scenes and variable datasets, addressing issues of missed and erroneous detections in classroom learning behavior recognition. Experimental evaluations reveal that our proposed method outperforms current mainstream one-stage and two-stage techniques in terms of accuracy. Additionally, ablation studies delve into the effectiveness of individual modules, laying a foundation for future enhancements.

In our future work, firstly, we will aim to reduce network parameters while ensuring detection accuracy, with the hope of creating a lightweight network that boasts high detection precision. This will cater to the real-time and lightweight requirements of classroom behavior recognition. Secondly, we intend to apply DMSA Net in various scenarios, such as lectures and meetings, to verify its effectiveness and generalization capabilities. Lastly, we will develop a system based on DMSA Net for recognizing and analyzing learning behaviors in natural classroom settings.

## Supplemental Information

10.7717/peerj-cs.2876/supp-1 Supplemental Information 1 Code.

## Additional Information and Declarations

### Data Availability

The following information was supplied regarding data availability:

The SCB-Dataset3-S is available at GitHub: https://github.com/Whiffe/SCB-dataset.

The self created object detection DataMountainSCB dataset is available at Github and Zenodo:

- https://github.com/Chunyu-Dong/DataFountainSCB1/tree/DataMountainSCb

- Chunyu-Dong. (2025). Chunyu-Dong/DataFountainSCB1: DataMountain SCBv1.0.0 (DataMountainSCb). Zenodo. https://doi.org/10.5281/zenodo.15009521.

## References

[ref-1] Abdallah TB, Elleuch I, Guermazi R (2021). Student behavior recognition in classroom using deep transfer learning with VGG-16. Procedia Computer Science.

[ref-2] Akçapınar G, Hasnine MN (2022). Discovering the effects of learning analytics dashboard on students’ behavioral patterns using differential sequence mining. Procedia Computer Science.

[ref-3] Andrade A, Delandshere G, Danish JA (2016). Using multimodal learning analytics to model student behavior: a systematic analysis of epistemological framing. Journal of Learning Analytics.

[ref-4] Bochkovskiy A, Wang CY, Liao HYM (2020). Yolov4: optimal speed and accuracy of object detection.

[ref-5] Cai Z, Vasconcelos N (2018). Cascade R-CNN: delving into high quality object detection.

[ref-6] Carreira J, Zisserman A (2017). Quo vadis, action recognition? a new model and the kinetics dataset.

[ref-7] Chen H, Zhou G, Jiang H (2023). Student behavior detection in the classroom based on improved YOLOv8. Sensors.

[ref-9] Gallagher J (2024). How to train an ultralytics YOLOv8 oriented bounding box (OBB) model. https://blog.roboflow.com/train-yolov8-obb-model/.

[ref-10] Gao J, Wei Y, Wang K, Shi Y (2023). Automatic academic emotion recognition and its evolution analysis in classroom.

[ref-11] Girshick R (2015). Fast R-CNN.

[ref-12] Girshick R, Donahue J, Darrell T, Malik J (2014). Rich feature hierarchies for accurate object detection and semantic segmentation.

[ref-13] Gowda SN (2017). Human activity recognition using combinatorial deep belief networks.

[ref-14] Gu F, Chung MH, Chignell M, Valaee S, Zhou B, Liu X (2021). A survey on deep learning for human activity recognition. ACM Computing Surveys (CSUR).

[ref-15] Gu A, Dao T (2023). Mamba: linear-time sequence modeling with selective state spaces.

[ref-16] Guo Q (2020). Detection of head raising rate of students in classroom based on head posture recognition. Traitement du Signal.

[ref-17] Hu M, Wei Y, Li M, Yao H, Deng W, Tong M, Liu Q (2022). Bimodal learning engagement recognition from videos in the classroom. Sensors.

[ref-18] Jisi A, Yin S (2021). A new feature fusion network for student behavior recognition in education. Journal of Applied Science and Engineering.

[ref-19] Krizhevsky A, Sutskever I, Hinton GE (2017). ImageNet classification with deep convolutional neural networks. Communications of the ACM.

[ref-20] Li X, Wang W, Hu X, Li J, Yang J (2021). Generalized focal loss V2: learning reliable localization quality estimation for dense object detection. IEEE.

[ref-21] Lin TY, Goyal P, Girshick R, He K, Dollár P (2017). Focal loss for dense object detection.

[ref-22] Liu W, Anguelov D, Erhan D, Szegedy C, Reed S, Fu CY, Berg ACS (2016). Single shot multibox detector.

[ref-23] Liu H, Ao W, Hong J (2021). Student abnormal behavior recognition in classroom video based on deep learning.

[ref-24] Liu Q, Jiang R, Xu Q, Wang D, Sang Z, Jiang X, Wu L (2024). YOLOv8n_BT: research on classroom learning behavior recognition algorithm based on improved YOLOv8n. IEEE Access.

[ref-25] Liu J, Jing D, Zhang H, Dong C (2024). SRFAD-Net: scale-robust feature aggregation and diffusion network for object detection in remote sensing images. Electronics.

[ref-26] Pishchulin L, Insafutdinov E, Tang S, Andres B, Andriluka M, Gehler PV, Schiele B (2016). DeepCut: joint subset partition and labeling for multi person pose estimation.

[ref-27] Redmon J, Divvala S, Girshick R, Farhadi A (2016). You only look once: unified, real-time object detection.

[ref-28] Redmon J, Farhadi A (2018). Yolov3: an incremental improvement.

[ref-8] Ren S, He K, Girshick R, Sun J (2017). Faster R-CNN: towards real-time object detection with region proposal networks. IEEE Transactions on Pattern Analysis & Machine Intelligence.

[ref-29] Ren X, Yang D (2021). Student behavior detection based on YOLOv4-Bi.

[ref-31] Sudhanan MS, Rahuman KA, Roomi MMS (2025). Using mask R-CNN and distance transform to detect elephants on railway tracks for collision prevention. Measurement.

[ref-32] Tang L, Xie T, Yang Y, Wang H (2022). Classroom behavior detection based on improved YOLOv5 algorithm combining multi-scale feature fusion and attention mechanism. Applied Sciences.

[ref-33] Tarasiou M, Chavez E, Zafeiriou S (2023). Vits for sits: vision transformers for satellite image time series.

[ref-34] Thuan D (2021). Evolution of Yolo algorithm and Yolov5: the state-of-the-art object detention algorithm. Bachelor’s thesis.

[ref-36] Van Etten A (2018). You only look twice: rapid multi-scale object detection in satellite imagery.

[ref-37] Wang CY, Bochkovskiy A, Liao HYM (2023). YOLOv7: trainable bag-of-freebies sets new state-of-the-art for real-time object detectors.

[ref-38] Wang A, Chen H, Liu L, Chen K, Lin Z, Han J (2024). YOLOV10: real-time end-to-end object detection. Advances in Neural Information Processing Systems.

[ref-39] Wang CY, Yeh IH, Mark Liao HY (2024). Yolov9: learning what you want to learn using programmable gradient information.

[ref-40] Wenchao L, Meng H, Yuping Z, Shuai L (2022). Research on intelligent recognition algorithm of college students’ classroom behavior based on improved SSD.

[ref-41] Yang F, Wang T (2023). SCB-Dataset3: a benchmark for detecting student classroom behavior.

[ref-42] Yang F, Wang T, Wang X (2023). Student classroom behavior detection based on YOLOv7+ BRA and multi-model fusion.

[ref-43] Yusuf A, Noor NM, Bello S (2024). Using multimodal learning analytics to model students’ learning behavior in animated programming classroom. Education and Information Technologies.

[ref-44] Zhang J, Lei J, Xie W, Fang Z, Li Y, Du Q (2023). SuperYOLO: super resolution assisted object detection in multimodal remote sensing imagery. IEEE Transactions on Geoscience and Remote Sensing.

[ref-45] Zhang Y, Wu Z, Chen X, Dai L, Li Z, Zong X, Liu T (2020). Classroom behavior recognition based on improved yolov3.

[ref-46] Zhao Y, Lv W, Xu S, Wei J, Wang G, Dang Q, Chen J (2024). DETRs beat YOLOs on real-time object detection.

[ref-47] Zhu W, Yang Z (2024). Csb-yolo: a rapid and efficient real-time algorithm for classroom student behavior detection. Journal of Real-Time Image Processing.

