# Supplementary figures and images for: DMSA-Net: a deformable multiscale adaptive classroom behavior recognition network
Source: PeerJ Comput Sci. 2025 Apr 30;11:e2876. doi: 10.7717/peerj-cs.2876 (PMC12192764; doi:10.7717/peerj-cs.2876)

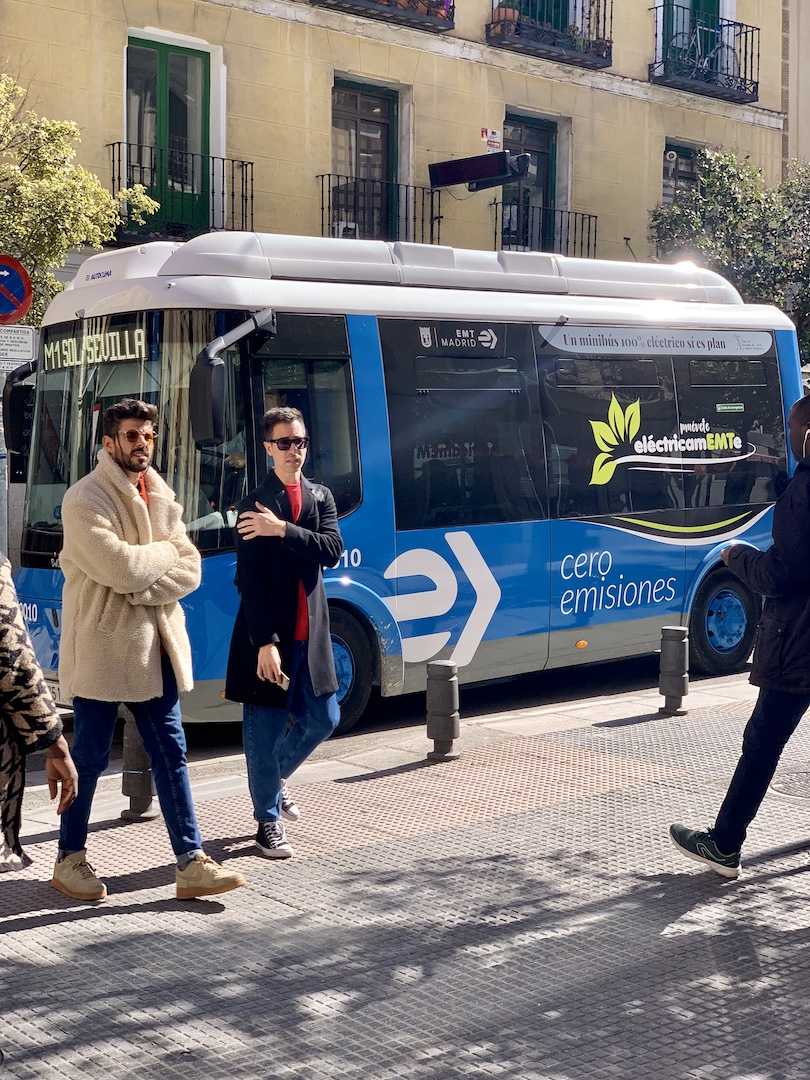

Supplement: Supplemental Information 1 [file peerj-cs-11-2876-s001.zip › ultralytics/assets/bus.jpg]

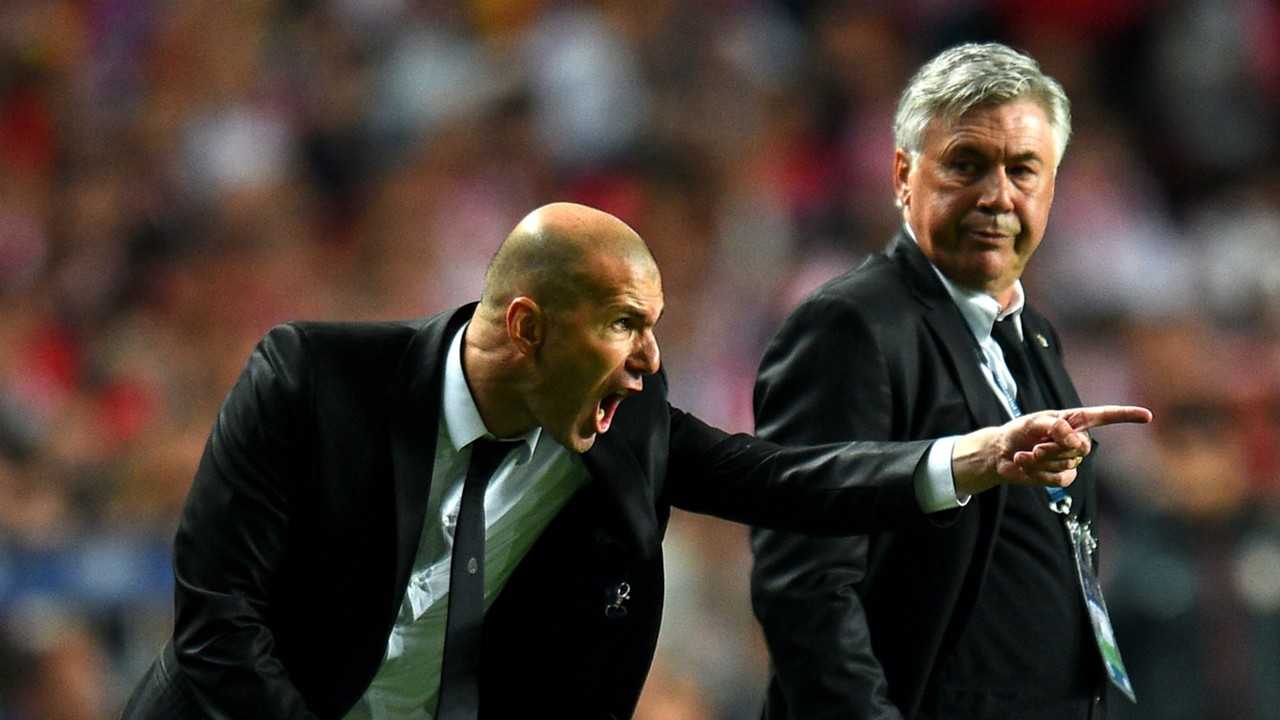

Supplement: Supplemental Information 1 [file peerj-cs-11-2876-s001.zip › ultralytics/assets/zidane.jpg]
